# Parent Preferences for Transparency of Their Child’s Hospitalization Costs

**DOI:** 10.1001/jamanetworkopen.2021.26083

**Published:** 2021-09-21

**Authors:** Hannah K. Bassett, Jimmy Beck, Ryan J. Coller, Brian Flaherty, Kristin A. Tiedt, Kevin Hummel, Michael J. Tchou, Kristopher Kapphahn, Lauren Walker, Alan R. Schroeder

**Affiliations:** 1 Division of Hospital Medicine, Department of Pediatrics, Stanford University School of Medicine, Stanford, California; 2 Department of Pediatrics, Seattle Children’s Hospital, Seattle, Washington; 3 Deparment of Pediatrics, University of Wisconsin School of Medicine and Public Health, Madison; 4 Division of Pediatric Critical Care, Department of Pediatrics, Primary Children’s Hospital, University of Utah, Salt Lake City; 5 currently affiliated with Division of Cardiology, Department of Pediatrics, Boston Children’s Hospital, Harvard Medical School, Boston, Massachusetts; 6 Division of Hospital Medicine, Department of Pediatrics, Cincinnati Children’s Hospital Medical Center, University of Cincinnati, Cincinnati, Ohio; 7 currently affiliated with Section of Hospital Medicine, Department of Pediatrics, Children’s Hospital Colorado, University of Colorado Denver, Aurora; 8 Quantitative Sciences Unit, Stanford University, Palo Alto, California; 9 Section of Hospital Medicine, Department of Pediatrics, Texas Children’s Hospital, Baylor College of Medicine, Houston; 10 Division of Critical Care, Department of Pediatrics, Stanford University School of Medicine, Palo Alto, California

## Abstract

**Importance:**

Health care in the US is often expensive for families; however, there is little transparency in the cost of medical services. The extent to which parents want cost transparency in their children’s care is not well characterized.

**Objective:**

To explore the preferences and experiences of parents of hospitalized children regarding the discussion and consideration of health care costs in the inpatient care of their children.

**Design, Setting, and Participants:**

This cross-sectional multicenter survey study included 6 geographically diverse university-affiliated US children’s hospitals from November 3, 2017, to November 8, 2018. Participants included a convenience sample of English- and Spanish-speaking parents of hospitalized children nearing hospital discharge. Data were analyzed from January 1, 2020, to June 25, 2021.

**Main Outcomes and Measures:**

Parents’ preferences and experiences regarding transparency of their child’s health care costs. Multivariable linear regression examined associations between clinical and sociodemographic variables with parents’ preferences for knowing, discussing, and considering costs in the clinical setting. Factors included family financial difficulties, child’s level of chronic disease, insurance payer, deductible, family poverty level, race, ethnicity, parental educational level, and study site.

**Results:**

Of 644 invited participants, 526 (82%) were enrolled (290 [55%] male), of whom 362 (69%) were White individuals, 400 (76%) were non-Hispanic/Latino individuals, and 274 (52%) had children with private insurance. Overall, 397 families (75%) wanted to discuss their child’s medical costs, but only 36 (7%) reported having a cost conversation. If cost discussions were to occur, 294 families (56%) would prefer to speak to a financial counselor. Ninety-eight families (19%) worried discussing costs would hurt the quality of their child’s care. Families with a medical financial burden unrelated to their hospitalized child had higher mean agreement that their child’s physician should consider the family’s costs in medical decision-making than families without a medical financial burden (effect size, 0.55 [95% CI, 0.18-0.92]). No variables were consistently associated with cost transparency preferences.

**Conclusions and Relevance:**

Most parents want to discuss their child’s costs during an acute hospitalization. Discussions of health care costs may be an important, relatively unexplored component of family-centered care. However, these discussions rarely occur, indicating a tremendous opportunity to engage and support families in this issue.

## Key Points

**Question:**

What are the preferences and experiences of parents regarding cost transparency in the care of their hospitalized child?

**Findings:**

In this cross-sectional survey study of 526 parents of hospitalized children, 398 (76%) believed it was important to know the costs of their child’s care, and 397 (75%) wanted a hospital employee to discuss these costs. However, only 36 parents (7%) reported having a cost discussion during admission.

**Meaning:**

These findings suggest that most families desire cost transparency in the care of their hospitalized child, but cost conversations rarely happen in the inpatient setting.

## Teaser

This cross-sectional survey study of parents of hospitalized children quantifies parental preferences, experiences, and barriers concerning cost transparency in hospital care.

## Introduction

Financial burden due to medical costs is common in the US,^1^ with 20% to 33% of adult patients reporting a medical financial burden^2,3,4,5^ and 16% to 50% of parents reporting a medical financial burden due to a child’s medical care.^6,7^ The economic constraints posed by the COVID-19 pandemic and ongoing threats to health care coverage could exacerbate these burdens.^8,9^ Previous studies have demonstrated that adult patients are amenable to cost discussions with their physicians.^10,11,12,13,14,15,16^ However, discussions of costs are infrequent in clinical encounters.^10,11,14,17,18,19^

Similar to adult care, spending on pediatric care is increasing.^20^ However, little research has examined parental preferences for transparency in their children’s costs of care.^21^ Although the American Academy of Pediatrics emphasizes the importance of sharing unbiased and comprehensive information with families, their policy statements on family-centered care do not specifically extend to cost information.^22,23^ To engage families on this critical issue, we need a better understanding of how parents regard cost transparency, which is defined for this study as both the discussion and consideration of a family’s costs (ie, the amount payable out-of-pocket for health care services^24^), in the clinical setting. In this multicenter study of parents of hospitalized children, our primary objective was to quantify parental preferences for, experiences with, and perceived barriers to cost transparency in the care of their hospitalized children. Our secondary objective was to identify associations between patient and family characteristics and cost transparency preferences.

## Methods

### Study Design and Population

This work was a planned analysis of a subset of data from our larger study, which also assessed financial difficulties in families of hospitalized children. The detailed methods of this study have been previously described.^25^ This cross-sectional survey study was conducted from November 3, 2017, to November 8, 2018, at 6 geographically diverse, university-affiliated children’s hospitals. The enrollment period at each site was variable, based on investigator availability and individual institutional board review approval. Participants were verbally informed of the research goals of the study and provided with a research information document. Need for written informed consent was determined by each institutional review board based on local research standards. Investigators at Primary Children’s Hospital obtained written informed consent, while the need for written informed consent was waived at the other institutions. The study followed the American Association for Public Opinion Research (AAPOR) reporting guidelines.

Study sites included Lucile Packard Children’s Hospital, Stanford, California; American Family Children’s Hospital, Madison, Wisconsin; Cincinnati Children’s Hospital and Medical Center, Cincinnati, Ohio; Primary Children’s Hospital, Salt Lake City, Utah; Seattle Children’s Hospital, Seattle, Washington; and Texas Children’s Hospital, Houston. Parents or guardians (hereafter referred to as parents) were eligible for enrollment if their child was admitted for any reason to 1 of the 6 hospitals during the enrollment period at that site. Exclusion criteria consisted of admission to a neonatal unit due to the confounding issue of mothers often being admitted concurrently, patient being 18 years of age or older (owing to possible incongruence between patient autonomy vs financial responsibility for care), parent being younger than 18 years, and language other than English or Spanish. Only 1 parent per child was eligible. Parents were approached by a study team member once their child had an anticipated discharge from the hospital within the next 48 hours. Each study site made an effort to enroll a consecutive sample of parents; however, this was limited by investigator availability. Surveys were completed on an electronic tablet at the time of enrollment. Participants at some sites (based on institutional research standards) were offered a $5 gift card as an incentive for survey completion. Each site had financial counselors available to families on request.

### Survey Instrument

The survey was developed by a multidisciplinary group that included experts in high-value care (A.R.S.) and administrators in hospital finance and billing. The survey was refined through review by local psychometric experts and members of the Family Advisory Council at the pilot research site to ensure appropriateness of content and reading level, leading to minor modifications. The final survey consisted of 40 items and was available in English and Spanish (translated by Idem Translations) (eAppendix in the Supplement). The survey included both adapted^10,12,15,26,27^ and de novo questions (eTable 1 in the Supplement). The survey was pilot tested by 10 parents (including English and Spanish speakers) to assess for adequate comprehension and clarity that did not inform any further changes. These data were not included in the final analysis.

### Measures

#### Outcomes

Our primary outcomes were parents’ cost transparency preferences, which were measured descriptively through survey items assessing their agreement with the importance of knowing, discussing, and considering their personal costs in their child’s medical care (items 1, 2, 4, and 6; eAppendix in the Supplement). These were answered on a 5-point Likert scale ranging from strongly disagree to strongly agree. Additional survey items provided descriptive context and detail to the outcomes described and included parents’ actual experience with discussing costs during their child’s hospitalization, concern about the cost of their child’s hospitalization, preferred timing and personnel for having cost discussions, and perceived barriers to having cost discussions.

#### Covariates

The level of chronic disease was categorized based on the consensus definitions from the Center of Excellence on Quality of Care Measures for Children with Complex Needs^28^ and assigned to each patient based on medical record review of problems and diagnoses listed in the electronic health record from the 3 years leading up to study enrollment, as available.^25^ Financial difficulties were defined by 2 separate measures. The first was the level of financial distress as defined by the InCharge Financial Distress/Financial Well-being Scale,^29^ which was included in our survey. The scale defines 3 categories of financial distress (high, average, and low) based on answers to 8 questions. The second measure of financial difficulty was the presence of a medical financial burden, which was categorized as being related to the hospitalized child, unrelated to the hospitalized child, or none, based on each parent’s subjective report on the survey. Insurance payer was defined as public or private based on the documented insurance plan in the electronic health record. Patients with dual public and private insurance were categorized as having public insurance based on the expertise of our financial administrators. Insurance deductible, parental educational level, and percentage of the federal poverty level were categorized based on parental report on the survey, whereas length of stay, stay in an intensive care unit during admission, race, and ethnicity were categorized based on documentation in the electronic health record. We collected both race and ethnicity as variables because we hypothesized that they may represent surrogates for cultural beliefs around health care decisions.

### Statistical Analysis

Data were analyzed from January 1, 2020, to June 25, 2021. The cooperation rate was calculated based on the AAPOR Cooperation Rate definition 4.^30^ Final survey responses were not weighted. Sample size calculations for this study were based on an outcome from our previously published study,^25^ where they are described in full. We aimed to enroll 150 participants at the pilot research site and 75 participants at the additional 5 research sites (Table 1). Relevant demographic and clinical variables, as well as our primary descriptive outcome measures, were summarized using tabulated frequencies. Responses of “I don’t know” were aggregated with responses of “no” when applicable. For our secondary analysis, we fit multivariable linear regression models to evaluate the association between our covariates and the responses to the 4 survey measures described. The Likert-scale responses were analyzed as a continuous variable, where positive effects represent stronger agreement and negative effects represent stronger disagreement with the survey question. Model diagnostic results, including residuals plots and q-q plots, were inspected to detect meaningful violations of linear regression model assumptions. All statistical analyses were conducted using R, version 3.5 (R Project for Statistical Computing).^31^ For the rare instances of missing clinical or sociodemographic data, the missing data were imputed using the mice package^32^ with 25 imputed data sets using 25 iterations each and pooled using the Rubin rules.^33^ All statistical tests were 2 sided and *P* < .05 indicated statistical significance.

**Table 1.** Characteristics of Parent Respondents and Their Hospitalized Children

Characteristic	Parent group^a^	
	Enrolled (n = 526)	Declined (n = 118)
Hospital		
Lucile Packard Children’s Hospital, Stanford, California	153 (29)	26 (22)
Seattle Children’s Hospital, Seattle, Washington	74 (14)	43 (36)
Primary Children’s Hospital, Salt Lake City, Utah	68 (13)	2 (2)
Texas Children’s Hospital, Houston	81 (15)	20 (17)
Cincinnati Children’s Hospital Medical Center, Cincinnati, Ohio	75 (14)	21 (18)
American Family Children’s Hospital, Madison, Wisconsin	75 (14)	6 (5)
Financial distress category		
High	125 (24)	NA
Average	262 (50)	NA
Low	139 (26)	NA
Medical financial burden		
Yes	160 (30)	NA
Related to hospitalized child (of those with any burden)	86 (54)	NA
No	361 (69)	NA
Missing	5 (1)	NA
Child’s chronic disease level^b^		
Complex	225 (43)	44 (37)
Noncomplex	143 (27)	58 (49)
None	157 (30)	16 (14)
Missing	1 (<1)	0
ICU during admission^b^		
Yes	90 (17)	8 (7)
No	435 (83)	107 (91)
Missing	1 (<1)	3 (3)
Length of stay, d^b^		
Median (IQR)	3 (2-7)	4 (2-10.75)
Missing	27 (5)	8 (7)
Child’s insurance^b^		
Public	244 (46)	68 (58)
Private	274 (52)	45 (38)
High deductible with private insurance (>$1000/y)^c^	163 (31)	0
Self-pay	1 (<1)	0
Unknown/missing	7 (1)	5 (4)
Ethnicity^b^		
Hispanic/Latino	111 (21)	18 (15)
Non-Hispanic/Latino	400 (76)	91 (77)
Unknown/missing	15 (3)	9 (8)
Race^b^		
American Indian/Alaska Native	4 (1)	2 (2)
Asian	36 (7)	16 (14)
Black	42 (8)	15 (13)
Native Hawaiian/Other Pacific Islander	5 (1)	0
White	362 (69)	62 (53)
Other^d^	62 (12)	14 (12)
Unknown/missing	15 (3)	9 (8)
Federal poverty level, %^e^		
≥400	157 (30)	NA
200-399	138 (26)	NA
100-199	90 (17)	NA
<100	92 (17)	NA
Prefer not to answer/missing	49 (9)	NA
Parental educational level (highest attained by either)^c^		
Less than high school	23 (4)	NA
High school/GED	156 (30)	NA
Degree		
Associate’s	106 (20)	NA
Bachelor’s	133 (25)	NA
Graduate	97 (18)	NA
Prefer not to answer/missing	11 (2)	NA

Abbreviations: GED, General Educational Development; IQR, interquartile range; ICU, intensive care unit; NA, not available.

^a^ Unless otherwise indicated, data are expressed as number (%) of respondents. Percentages have been rounded and may not total 100.

^b^ Abstracted from the electronic health record.

^c^ Self-reported.

^d^ Represents a distinct category for race as obtained from the electronic health record.

^e^ Calculated value based on reported annual household income and number of individuals living in the household.

## Results

Of 644 parents who were approached, 523 returned complete surveys, and 3 returned partial surveys (cooperation rate of 82%), with a final sample of 526 (290 [55%] male and 236 [45%] female). Most of our sample categorized as White (362 [69%]) and non-Hispanic/Latino (400 [76%]). More than half of children had private insurance (274 [52%]). Table 1 lists the demographic characteristics of the enrolled patients and parents (including missing data) and nonresponders. Clinical and demographic characteristics by study site are shown in eTable 2 in the Supplement. There were no missing data for our primary outcomes.

**Figure 1.**
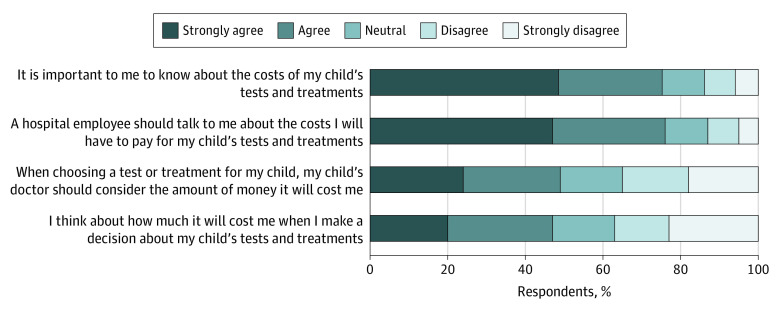
Parental Preferences on Knowing, Discussing, and Considering Their Child’s Health Care Costs Includes 526 parent respondents.

Most parents (398 [76%]) strongly agreed or agreed that knowing the costs of their child’s care was important (Figure 1). Similarly, most parents (397 [75%]) strongly agreed or agreed that a hospital employee should talk to them about the costs they will have to pay for their child’s care. In comparison, almost half of parents (259 [49%]) strongly agreed or agreed that the physician should consider the parent’s costs when making medical decisions for their child, with 185 parents (35%) disagreeing or strongly disagreeing. In addition, almost half of parents (250 [48%]) strongly agreed or agreed that they considered their own costs when making medical decisions for their child, with 193 (37%) disagreeing or strongly disagreeing. Most parents (397 [75%]) reported they were concerned about how much their child’s hospitalization would cost them personally, with 169 (32%) being moderately or very concerned. Descriptive results stratified by study site are shown in eTable 3 in the Supplement.

**Figure 2.**
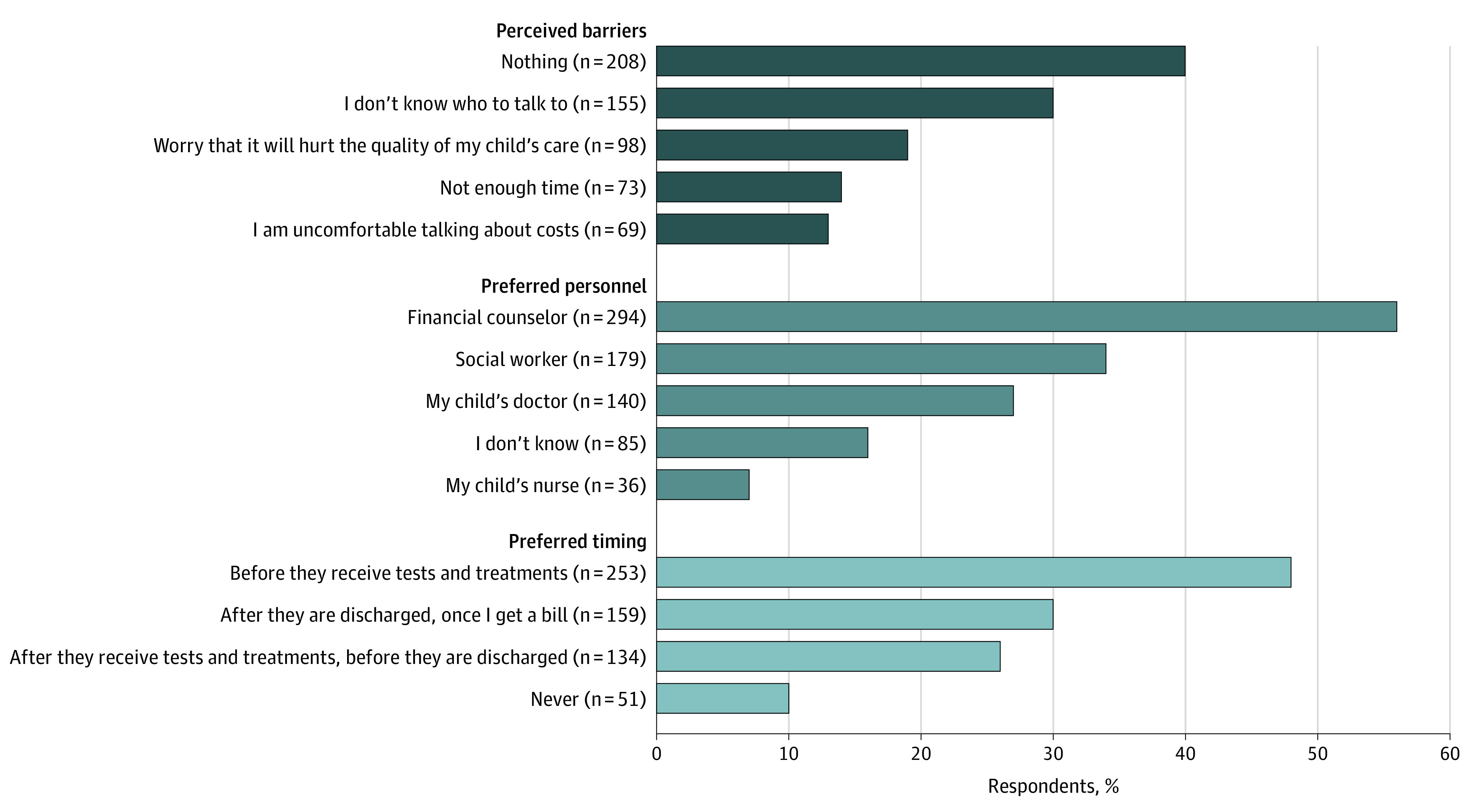
Parents’ Perceived Barriers to and Detailed Preferences for Cost Discussions During Their Child’s Hospitalization Includes 524 parent respondents. Percentages may total more than 100 because respondents were instructed to check all that applied.

In contrast, only 165 parents (31%) recalled a specific time during their child’s hospitalization when they wanted to discuss costs. Of those parents, only 36 (22%, representing 7% of the total sample) had a conversation of that nature. Although 208 respondents (40%) stated that nothing would prevent them from discussing costs, others perceived barriers (Figure 2). Ninety-eight families (19%) worried discussing costs would hurt the quality of their child’s care. If these conversations were to occur, 294 parents (56%) preferred a financial counselor to be the source of information. Finally, 253 parents (48%) indicated that they would want to have cost conversations before their child received tests and treatments. Only 51 parents (10%) indicated they would never want to have these conversations.

No independent variables had a significant association with all cost transparency preference measures (Table 2). Families with a medical financial burden unrelated to the hospitalized child had higher mean agreement that their child’s physician should consider the family’s costs (effect size, 0.55 [95% CI, 0.18-0.92]; *P* = .004) compared with families with no medical financial burden. However, neither measure of financial difficulty had any additional association with cost transparency preferences. Parents whose child was in an intensive care unit during their admission had higher mean agreement that it was important to know their child’s costs of care compared with families whose child was not in an intensive care unit (effect size, 0.30 [95% CI, 0.02-0.58]). Study site was associated with the 2 survey measures regarding considering costs in medical decision-making, notably with parents at Primary Children’s Hospital having higher mean agreement with both measures (effect sizes, 0.86 [95% CI, 0.42-1.30] for the child’s physician to consider costs when choosing a treatment and 0.57 [95% CI, 0.11-1.02] for thinking about costs when making a decision about treatment). Race was associated with parents thinking about costs when making a decision about treatment (effect size, 0.66 [95% CI, 0.15-1.18]), and compared with parents who had not finished high school, all parental educational levels were negatively associated with wanting the child’s physician to consider costs in medical decision-making (eg, graduate degree; effect size −1.06 [95% CI, −1.79 to −0.33]). No other variable had statistically significant associations with responses on any survey measure (Table 2).

**Table 2.** Multivariable Linear Regression Modeling the Effect of Clinical and Sociodemographic Variables on 4 Measures of Parental Cost Transparency Preferences

Variable	Measure, effect size (95% CI)^a^			
	It is important to me to know about the costs of my child’s tests and treatments.	A hospital employee should talk to me about the costs I will have to pay for my child’s tests and treatments.	When choosing a test or treatment for my child, my child’s doctor should consider the amount of money it will cost me.	I think about how much it will cost me when I make a decision about my child’s tests and treatments.
Financial distress				
Low	1 [Reference]	1 [Reference]	1 [Reference]	1 [Reference]
Average	0.12 (−0.15 to 0.40)	0.11 (−0.16 to 0.38)	0.14 (−0.18 to 0.46)	0.30 (−0.03 to 0.63)
High	0.20 (−0.17 to 0.57)	0.13 (−0.24 to 0.50)	−0.04 (−0.47 to 0.40)	0.19 (−0.26 to 0.65)
*P* value	.55	.70	.45	.19
Medical financial burden				
None	1 [Reference]	1 [Reference]	1 [Reference]	1 [Reference]
Child related	−0.05 (−0.37 to 0.28)	−0.09 (−0.40 to 0.23)	−0.16 (−0.54 to 0.21)	−0.08 (−0.47 to 0.32)
Child unrelated	0.32 (0.00 to 0.64)	0.34 (0.03 to 0.65)	0.55 (0.18 to 0.92)	0.30 (−0.09 to 0.68)
*P* value	.10	.06	.004	.23
Child’s level of chronic disease				
None	1 [Reference]	1 [Reference]	1 [Reference]	1 [Reference]
Noncomplex	0.14 (−0.18 to 0.45)	0.10 (−0.21 to 0.40)	0.22 (−0.15 to 0.58)	0.29 (−0.09 to 0.67)
Complex	0.06 (−0.20 to 0.32)	−0.06 (−0.31 to 0.19)	0.13 (−0.17 to 0.44)	0.01 (−0.31 to 0.32)
*P* value	.69	.54	.48	.22
ICU during admission				
No	1 [Reference]	1 [Reference]	1 [Reference]	1 [Reference]
Yes	0.30 (0.02 to 0.58)	0.27 (0.00 to 0.55)	0.18 (−0.14 to 0.51)	−0.09 (−0.43 to 0.25)
*P* value	.04	.05	.27	.60
Length of stay, wk				
Continuous	0.00 (−0.02 to 0.03)	−0.01 (−0.03 to 0.02)	0.01 (−0.01 to 0.04)	0.00 (−0.02 to 0.03)
*P* value	.68	.56	.34	.75
Child’s insurance payer^b^				
Private	1 [Reference]	1 [Reference]	1 [Reference]	1 [Reference]
Public	−0.05 (−0.39 to 0.30)	0.05 (−0.29 to 0.39)	−0.05 (−0.46 to 0.35)	0.06 (−0.37 to 0.48)
*P* value	.73	.72	.73	.72
Deductible >$1000/y				
No	1 [Reference]	1 [Reference]	1 [Reference]	1 [Reference]
Yes	0.10 (−0.25 to 0.44)	0.21 (−0.12 to 0.53)	0.24 (−0.17 to 0.65)	0.15 (−0.25 to 0.56)
*P* value	.57	.21	.25	.43
Ethnicity				
Non- Hispanic/Latino	1 [Reference]	1 [Reference]	1 [Reference]	1 [Reference]
Hispanic/Latino	−0.27 (−0.58 to 0.05)	−0.21 (−0.52 to 0.09)	−0.17 (−0.53 to 0.20)	−0.10 (−0.49 to 0.28)
*P* value	.10	.18	.37	.59
Race^c^				
White	1 [Reference]	1 [Reference]	1 [Reference]	1 [Reference]
Asian	0.38 (−0.04 to 0.80)	0.32 (−0.09 to 0.74)	0.27 (−0.22 to 0.77)	0.66 (0.15 to 1.18)
Black	0.27 (−0.15 to 0.69)	0.28 (−0.13 to 0.68)	0.04 (−0.44 to 0.53)	0.49 (−0.02 to 0.99)
Other	0.25 (−0.12 to 0.62)	−0.03 (−0.40 to 0.33)	0.18 (−0.26 to 0.61)	0.24 (−0.21 to 0.69)
*P* value	.14	.25	.63	.02
Federal poverty level, %				
≥400	1 [Reference]	1 [Reference]	1 [Reference]	1 [Reference]
200-399	0.01 (−0.29 to 0.32)	0.08 (−0.23 to 0.38)	0.31 (−0.05 to 0.67)	0.40 (0.02 to 0.78)
100-199	−0.09 (−0.48 to 0.31)	−0.06 (−0.46 to 0.34)	0.18 (−0.29 to 0.65)	0.22 (−0.27 to 0.70)
<100	−0.11 (−0.56 to 0.33)	−0.11 (−0.55 to 0.34)	0.50 (−0.05 to 1.04)	0.26 (−0.30 to 0.82)
*P* value	.87	.72	.17	.19
Parental educational level				
Less than high school	1 [Reference]	1 [Reference]	1 [Reference]	1 [Reference]
High school/GED	−0.54 (−1.08 to 0.00)	−0.22 (−0.75 to 0.30)	−1.05 (−1.67 to −0.42)	−0.61 (−1.26 to 0.04)
Degree				
Associate’s	−0.69 (−1.27 to −0.12)	−0.41 (−0.97 to 0.15)	−1.30 (−1.96 to −0.64)	−0.71 (−1.40 to −0.02)
Bachelor’s	−0.76 (−1.35 to −0.16)	−0.57 (−1.16 to 0.01)	−1.25 (−1.94 to −0.56)	−0.69 (−1.41 to 0.03)
Graduate	−0.53 (−1.15 to 0.10)	−0.20 (−0.81 to 0.42)	−1.06 (−1.79 to −0.33)	−0.60 (−1.36 to 0.16)
*P* value	.09	.06	.003	.34
Study site				
Lucile Packard Children’s Hospital, Stanford, California	1 [Reference]	1 [Reference]	1 [Reference]	1 [Reference]
American Family Children’s Hospital, Madison, Wisconsin	−0.19 (−0.56 to 0.18)	−0.20 (−0.56 to 0.16)	−0.10 (−0.53 to 0.33)	−0.20 (−0.64 to 0.25)
Cincinnati Children’s Hospital and Medical Center, Cincinnati, Ohio	−0.13 (−0.50 to 0.25)	−0.32 (−0.69 to 0.04)	−0.52 (−0.95 to −0.08)	−0.37 (−0.82 to 0.08)
Primary Children’s Hospital, Salt Lake City, Utah	−0.01 (−0.38 to 0.37)	−0.05 (−0.42 to 0.32)	0.86 (0.42 to 1.30)	0.57 (0.11 to 1.02)
Seattle Children’s Hospital, Seattle, Washington	−0.39 (−0.78 to 0.01)	−0.38 (−0.77 to 0.01)	0.25 (−0.21 to 0.72)	0.15 (−0.33 to 0.63)
Texas Children’s Hospital, Houston	0.23 (−0.14 to 0.59)	−0.04 (−0.40 to 0.31)	0.27 (−0.15 to 0.69)	0.12 (−0.32 to 0.55)
*P* value	.13	.27	<.001	.007

Abbreviations: GED, General Educational Development; ICU, intensive care unit.

^a^ Positive effect sizes represent increased “agreement” on the Likert scale response for each survey measure, whereas negative effect sizes represent increased “disagreement” on the Likert scale response. *P* values were derived from type 3 F tests. Values of unknown or prefer not to answer were treated as missing, and these values were imputed.

^b^ Self-payer was included in the category of private to ensure sufficient cell sizes for modeling.

^c^ American Indian/Alaska Native and Native Hawaiian/Other Pacific Islander were included in the category of other (a distinct category for race as obtained from the electronic health record) to ensure sufficient cell sizes for modeling.

## Discussion

In this multicenter study, we found that most parents believe it is important to know about their child’s costs of care and want to discuss these costs during hospitalization. However, only a small minority of parents reported actually having a discussion regarding these costs. Perceived barriers to cost discussions were not knowing who to talk to and fear of affecting the quality of their child’s care.

Most families agreed that someone should speak to them about their personal costs for their child’s care, with almost half of parents strongly agreeing compared with only 5% strongly disagreeing. These results are similar to previously published adult studies.^12,14,15,34^ Notably, much of the adult literature has focused on patients with cancer,^11^ the treatment of which can impose severe financial hardships on patients. Our results suggest that health care costs are an equally important topic, even among a more heterogenous and less financially strained population, and therefore may be an important component of family-centered care.

Family-centered care fosters the engagement of families in their children’s medical care by respecting family values, building collaborative relationships between families and the health care team, and providing transparent and comprehensive health information.^35^ Although cost transparency has not been part of the traditional family-centered care model, our results suggest that perhaps it should be. Family-centered cost transparency will need to be thoughtfully implemented in this clinical setting because acute hospitalization has multiple compounding psychosocial and financial stressors. However, facilitating access to costs during hospitalization may provide families with early clarity and support for their financial responsibility for care, and our results provide some early considerations.

Despite widespread interest, cost discussions rarely occurred. This discrepancy has been demonstrated frequently in the adult literature as well.^10,11,14,36^ The feasibility of making these conversations standard of care is limited by challenges in providing timely and accurate cost estimates to physicians and patients. In addition, the culture within our health care system is to separate the clinical and financial aspects of care. Despite most of our participants indicating that knowing costs is important, fewer than one-third recalled a specific instance during their child’s hospitalization when they wanted to discuss costs. This discrepancy could reflect the lack of precedent for having financial discussions at the point of care; parents may not have thought about discussing costs because they never have before.

Similar to the findings of Beck et al,^21^ families in our study were more open to having these conversations with nonclinical individuals. Furthermore, 19% of our population had concerns that cost transparency may negatively affect the quality of their child’s care, highlighting how some parents may prefer distancing discussions of cost from medical decision-making. Models that support and use trained financial navigators, such as those piloted in some ambulatory settings,^37,38,39^ may be successful in the inpatient setting. Including financial staff in the multidisciplinary team could help families receive cost information without concerns about disrupting the therapeutic alliance with medical professionals.

However, some parents indicated they want their personal costs incorporated into medical decision-making. Almost half of parents indicated they consider their own costs or want their child’s physician to consider their costs when making medical decisions. These percentages are somewhat higher than results from adult studies.^10,15,40^ Finances are likely an important contributor to a parent’s sense of responsibility to care for their children and may factor prominently as they balance their child’s health with the needs of their family. Although we do not suggest a family’s costs should be the sole consideration in medical decisions, we do believe costs could be an important part of a comprehensive discussion with interested families regarding risk and benefit trade-offs when clinically appropriate.

Our multivariable regression model demonstrated several associations. Study site was significantly associated with 2 measures of cost transparency. This is a potentially important finding that may reflect differences in cost of living, sociopolitical variations, or nuances within the medical culture at each institution. However, further research is needed to understand the underlying factors for these associations. In addition, families that had a medical financial burden unrelated to their child had a higher mean agreement that costs should be considered by their child’s physician. These families likely have a heightened awareness of medical costs. This association was not seen in families with a child-related medical financial burden, potentially resulting from discrepancies in insurance between parents and children leading to different expectations for hospital bills and financial responsibility. Having a child in the intensive care unit was positively associated with wanting to know costs, but not with discussing costs or considering costs in decision-making. Parents are likely aware that the intensive care unit is expensive, which may be the reason for their preference to know costs, but the anxiety of having a critically ill child may affect their desire to actually discuss or consider costs in their care. Finally, we hypothesized that race and ethnicity may represent surrogate markers for cultural beliefs around health care decisions and therefore may contribute to parents’ preferences for cost transparency. These variables were not consistently or strongly associated with cost transparency preferences; however, we believe this remains an important area for further exploration to ensure patient-level cost transparency does not reinforce structural biases or the marginalization of patient populations.

Although some of the barriers to family-centered cost transparency will require national reform, individual organizations can take steps to foster these conversations. Families can be screened for financial concerns on admission, similar to screening for other unmet social needs, and institutions can implement pathways for addressing these concerns. Hospitals can develop robust, accessible financial services that proactively contact all families to provide transparency about billing processes and financial assistance programs. Finally, curricula focused on costs of care and health care financing could be routinely implemented across the medical education spectrum to bridge the gap between the clinical and financial aspects of care.

### Limitations

We acknowledge several limitations of this study. First, the terms used to describe consumer health care expenditures, such as *cost*, *price*, *charge*, and *fee*, are often used inconsistently and interchangeably with varying interpretations across stakeholders. We chose to use *cost* because this has been the precedent in previous literature on this topic. Second, we surveyed families before hosptial discharge and therefore before the receipt of any hospital bills. Families’ perspectives on the importance of discussing costs could change once they have the opportunity to review their personal financial responsibility for the hospitalization. However, we did not find any association between level of chronic disease and cost transparency preferences, despite the likelihood that families of children with chronic disease would have experience with previous hospital bills. Third, our sample consisted largely of non-Hispanic, White, and English-speaking individuals and therefore may not reflect the general population. In addition, race and ethnicity were obtained through documentation in the electronic health record as opposed to direct self-report, potentially leading to misclassification. Fourth, although we had geographic diversity in our sample, our population was recruited from academic children’s hospitals and therefore may be more difficult to generalize to all families. Last, our study was initially powered to detect differences in the financial distress of our participants,^25^ and the small sizes of some of the subgroups in our analysis signify that we may have been underpowered to detect small but potentially important differences in cost transparency preferences.

## Conclusions

Most parents, regardless of clinical or demographic characteristics, want to discuss their child’s costs during an acute hospitalization, yet these conversations happen rarely. Discussions of health care cost may be an important, relatively unexplored component of family-centered care. Health systems, hospital administrators, and physicians should be proactive in developing strategies to facilitate early access to health care costs to better engage and support families in this crucial topic.

## Notes

Supplement. **eAppendix.** Complete Survey Tool Provided to Parents **eTable 1.** Survey Domains, Measures, and References **eTable 2.** Characteristics of Parent Respondents and Their Hospitalized Children Stratified by Study Site **eTable 3.** Parental Preferences on Knowing, Discussing, and Considering Their Child’s Health Care Costs, Stratified by Study Site
eReferences.
