# Supplementary material for: Parent Preferences for Transparency of Their Child’s Hospitalization Costs
Source: JAMA Netw Open. 2021 Sep 21;4(9):e2126083. doi: 10.1001/jamanetworkopen.2021.26083 (PMC8456391; doi:10.1001/jamanetworkopen.2021.26083)
Supplement: Supplement. — eAppendix. Complete Survey Tool Provided to Parents eTable 1. Survey Domains, Measures, and References eTable 2. Characteristics of Parent Respondents and Their Hospitalized Children Stratified by Study Site eTable 3. Parental Preferences on Knowing, Discussing, and Considering Their Child’s Health Care Costs, Stratified by Study Site eReferences. [file jamanetwopen-e2126083-s001.pdf]

Supplementary Online Content

Bassett HK, Beck J, Coller RJ, et al. Parent preferences for transparency of their child's hospitalization costs. *JAMA Netw Open*. 2021;4(9):e2126083.
doi:10.1001/jamanetworkopen.2021.26083

eAppendix. Complete Survey Tool Provided to Parents

eTable 1. Survey Domains, Measures, and References

eTable 2. Characteristics of Parent Respondents and Their Hospitalized Children Stratified by Study Site

eTable 3. Parental Preferences on Knowing, Discussing, and Considering Their Child's Health Care Costs, Stratified by Study Site

eReferences.

This supplementary material has been provided by the authors to give readers additional information about their work.

©2021 Bassett HK et al. *JAMA Network Open*.

eAppendix. Complete Survey Tool Provided to Parents

For this survey:

- all questions refer to your child who is currently in the hospital
- "hospital employee" could be a healthcare professional (for example, a doctor or nurse), a social worker, a financial counselor, or other administrator
- "tests and treatments" refers to any medical service that your child might receive while in the hospital

The following questions will ask about your thoughts about the healthcare costs of your child. There are no right or wrong answers. These questions are for research purposes only. Your answers will have no impact on your child's current hospitalization or care. Your answers will not result in immediate changes in the way hospital employees share information about healthcare costs, but might change how they talk about costs with parents in the future.

Please rate how much you agree or disagree with the next statements.

1. It is important to me to know about the costs of my child's tests and treatments.
    a. Strongly agree
    b. Agree
    c. Neither agree nor disagree
    d. Disagree
    e. Strongly disagree

2. A hospital employee should talk to me about the costs I will have to pay for my child's tests and treatments.
    a. Strongly agree
    b. Agree
    c. Neither agree nor disagree
    d. Disagree
    e. Strongly disagree

3. A hospital employee should talk to me about the costs society (the insurance company, government) will have to pay for my child's tests and treatments.
    a. Strongly agree
    b. Agree
    c. Neither agree nor disagree
    d. Disagree
    e. Strongly disagree

4. When choosing a test or treatment for my child, my child's doctor should consider the amount of money it will cost me.
    a. Strongly agree
    b. Agree
    c. Neither agree nor disagree
    d. Disagree
    e. Strongly disagree

5. When choosing a test or treatment for my child, my child's doctor should consider the amount of money it will cost society (the insurance company, government).
    a. Strongly agree
    b. Agree
    c. Neither agree nor disagree
    d. Disagree
    e. Strongly disagree

6. I think about how much it will cost me when I make a decision about my child's tests and treatments.
    a. Strongly agree
    b. Agree
    c. Neither agree nor disagree
    d. Disagree
    e. Strongly disagree

©2021 Bassett HK et al. *JAMA Network Open*.

7. I think about how much it will cost <u>society</u> (the insurance company, government) when I make a decision about my child's tests and treatments.
    a. Strongly agree
    b. Agree
    c. Neither agree nor disagree
    d. Disagree
    e. Strongly disagree

8. (Randomized with 9) A hospital that charges more than another hospital for the same test or treatment is probably providing higher quality care.
    a. Strongly agree
    b. Agree
    c. Neither agree nor disagree
    d. Disagree
    e. Strongly disagree

9. A hospital that charges less than another hospital for the same test or treatment is probably providing lower quality care.
    a. Strongly agree
    b. Agree
    c. Neither agree nor disagree
    d. Disagree
    e. Strongly disagree

10. (Randomized with 11) Treatments that work better usually cost more than treatments that don't work as well.
    a. Strongly agree
    b. Agree
    c. Neither agree nor disagree
    d. Disagree
    e. Strongly disagree

11. Treatments that do not work as well usually cost less than treatments that work better.
    a. Strongly agree
    b. Agree
    c. Neither agree nor disagree
    d. Disagree
    e. Strongly disagree

12. (Randomized with 13) Tests that are more accurate usually cost more than tests that are less accurate.
    a. Strongly agree
    b. Agree
    c. Neither agree nor disagree
    d. Disagree
    e. Strongly disagree

13. Tests that are less accurate usually cost less than tests that are more accurate.
    a. Strongly agree
    b. Agree
    c. Neither agree nor disagree
    d. Disagree
    e. Strongly disagree

14. How concerned are you about how much your child's hospitalization will cost you?
    a. Not at all concerned
    b. Slightly concerned
    c. Somewhat concerned
    d. Moderately concerned
    e. Very concerned

©2021 Bassett HK et al. *JAMA Network Open*.

15. During your child’s hospitalization, have there been times when you wanted to talk about the costs of your child’s tests and treatments with a hospital employee?
    a. Yes
    b. No

16. Did you talk about the costs of your child’s tests and treatments with a hospital employee?
    a. Yes
    b. No

17. What would keep you from starting a discussion about the costs of your child’s tests and treatments with a hospital employee? Check all that apply.
    a. Nothing would keep me from starting a discussion
    b. Not enough time
    c. Worry that discussing costs would hurt the quality of my child’s care
    d. I don’t know who to talk to about their costs
    e. I am not comfortable talking about their costs
    f. Other: ______________________

18. Who would you want to talk to you about the costs of your child’s tests and treatments? Check all that apply.
    a. My child’s doctor
    b. My child’s nurse
    c. A financial counselor
    d. A social worker
    e. Other: _______________________
    f. I don’t know

19. When would you want to talk to a hospital employee about the costs of your child’s tests and treatments? Check all that apply.
    a. Before they get their tests and treatments
    b. After they get their tests and treatments, but before they are discharged from the hospital
    c. After they are discharged from the hospital, once I get a bill
    d. Never
    e. Other:________________________

20. Did you know that this hospital has financial counselors?
    a. Yes
    b. No

21. Did you ask to speak with a financial counselor during your child’s hospitalization?
    a. Yes
    b. No

22. Why did you ask to speak with a financial counselor?
    a. To sign up for emergency insurance coverage
    b. To ask a question about my current insurance coverage
    c. To ask about financial assistance policies
    d. To get an estimate of what I will be responsible for paying
    e. Other: ___________________

The following questions will ask about your child’s health insurance. These questions are for research purposes only, and meant to help us understand what type of health insurance plan your child is enrolled in. Your answers will have no impact on your child’s current hospitalization or their insurance coverage.

1. Is your child’s health insurance Medicaid?
    a. Yes
    b. No

2. Does your child's insurance plan have a deductible? A deductible is the amount you have to pay for medical services before your child's health insurance starts to pay.
    a. Yes
    b. No
    c. I don't know

1a. How much is your child's insurance deductible each year?
    a. Less than $500
    b. $500-$1000
    c. $1001 to $3000
    d. More than $3000
    e. I do not know

1b. Before to this hospitalization, had your child met their insurance deductible for the year? In other words, had your child's medical bills added up to more than the amount of their deductible?
    a. Yes
    b. No
    c. I don't know

3. Does your child's insurance plan require a copayment for hospital services? A copayment is a fixed fee you pay when your child receives a certain type of medical service.
    a. Yes
    b. No
    c. I don't know

4. Does your child's insurance plan require coinsurance for hospital services? Coinsurance is a percentage of the total hospital charges you are required to pay after you have met your child's insurance deductible.
    a. Yes
    b. No
    c. I don't know

The following questions will ask about how you feel about your current financial situation. These questions are for research purposes only, and are meant to help us understand what factors contribute to how parents view healthcare costs. If you have financial concerns regarding your child's hospitalization, you should contact this hospital's Financial Assistance Department.

1. Do you or your spouse/partner (if applicable) have any current financial burden from medical care? Financial burden could include: difficulty paying or inability to pay medical bills, using personal savings to pay medical bills, difficulty paying for food, gas, or rent because of medical bills, declaring bankruptcy because of medical bills, etc.
    a. Yes
    b. No
    c. I don't know

1a. Is your current financial burden due to your hospitalized child's medical care?
    a. Yes
    b. No

InCharge Financial Distress/Financial Well-Being Scale© (© by InCharge Education Foundation and E. Thomas Garman, 2005. All rights reserved)

Directions: Check the responses that are ***most appropriate*** for your situation.

1. What do you feel is the ***level*** or your ***financial stress today?***

1	2	3	4	5	6	7	8	9	10
Overwhelming Stress			High Stress		Low Stress		No Stress at All		

2. On the stair steps below, mark how ***satisfied*** you are with your ***present financial situation.*** The "1" at the bottom of the steps represents complete dissatisfaction. The "10" at the top if the steps represents complete satisfaction. The more dissatisfied you are, the lower the number you should mark. The more satisfied you are, the higher the number you should mark.

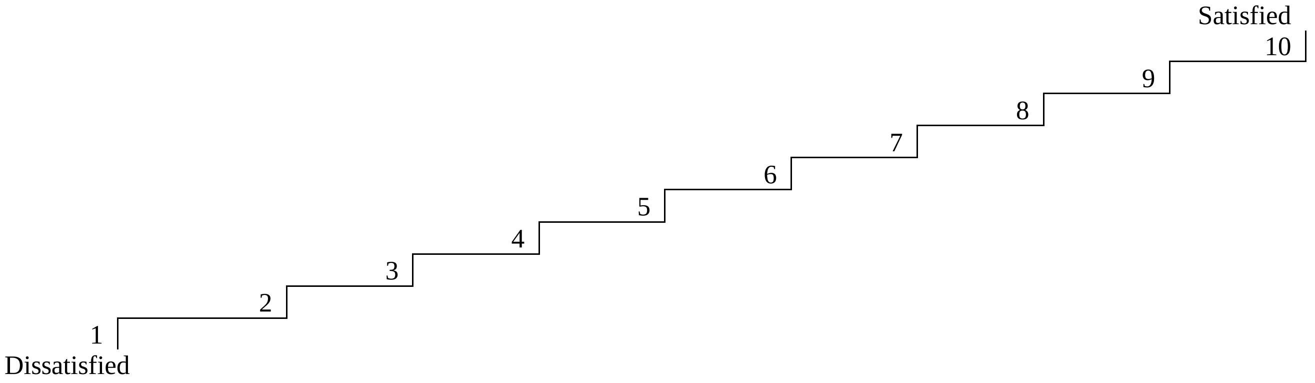

3. How do you feel about your ***current financial situation?***

1	2	3	4	5	6	7	8	9	10
Feel Overwhelmed			Sometimes Feel Worried		Not Worried		Feel Comfortable		

4. How often do you worry about being ***able to meet*** normal monthly living expenses?

1	2	3	4	5	6	7	8	9	10
Feel Overwhelmed			Sometimes Feel Worried		Not Worried		Feel Comfortable		

5. How confident are you that you could find the money to pay for a **financial emergency** that costs about $1,000?

1	2	3	4	5	6	7	8	9	10
No Confidence			Little Confidence		Some Confidence		High Confidence		

6. How often does this happen to you? You want to go out to eat, go to a movie, or do something else and ***don't go because you can't afford to?***

1	2	3	4	5	6	7	8	9	10
All the time			Sometimes		Rarely		Never		

7. How frequently do you find yourself just getting by financially and living **paycheck to paycheck?**

1	2	3	4	5	6	7	8	9	10
All of the time			Sometimes		Rarely		Never		

8. How **stressed** do you feel about your personal finances **in general?**

1	2	3	4	5	6	7	8	9	10
Overwhelming Stress			High Stress		Low Stress		No Stress at All		

1. What is your relationship to your child?
   a. Mother/Female guardian
   b. Father/Male guardian
   c. Other: ___________________

2. What is your age?

©2021 Bassett HK et al. *JAMA Network Open*.

a. 18-29 years old
b. 30-49 years old
c. 50-64 years old
d. 65 year old or older

3. What is the highest level of education you or your partner/spouse (if applicable) have completed?
    a. Less than high school
    b. High school degree or equivalent, (for example, GED)
    c. Vocational or Associate's degree (for example, AA, AS)
    d. Bachelor's degree (for example, BA, BS)
    e. Professional or graduate degree (for example, MA, MD, DDS, PhD)
    f. Prefer not to answer

4. What is your total yearly household income before taxes?
    a. Less than $25,000
    b. $25,000-$49,999
    c. $50,000-$74,999
    d. $75,000-$99,999
    e. $100,000-$199,999
    f. $200,000-$299,999
    g. More than $300,000
    h. Prefer not to answer

5. Including yourself, how many people (adults and children) does this household income support?
    a. Number of people: _____
    b. Prefer not to answer

©2021 Bassett HK et al. *JAMA Network Open*.

eTable 1. Survey Domains, Measures, and References

Domain	Survey measure	Reference measure was adapted from
**Personal cost transparency preferences**	It is important to me to know about the costs of my child's tests and treatments.	De novo
	A hospital employee should talk to me about the costs I will have to pay for my child's tests and treatments.	1
	When choosing a test or treatment for my child, my child's doctor should consider the amount of money it will cost me.	
	I think about how much it will cost me when I make a decision about my child's tests and treatments.	2
	What would keep you from starting a discussion about the costs of your child's tests and treatments with a hospital employee? Check all that apply.	1
	Who would you want to talk to you about the costs of your child's tests and treatments? Check all that apply.	2
	When would you want to talk to a hospital employee about the costs of your child's tests and treatments? Check all that apply.	
**Social cost transparency preferences**	A hospital employee should talk to me about the costs society (the insurance company, government) will have to pay for my child's tests and treatments.	1
	When choosing a test or treatment for my child, my child's doctor should consider the amount of money it will cost society (the insurance company, government).	
	I think about how much it will cost society (the insurance company, government) when I make a decision about my child's tests and treatments.	2
**Experience**	How concerned are you about how much your child's hospitalization will cost you?	De novo
	During your child's hospitalization, have there been times when you wanted to talk about the costs of your child's tests and treatments with a hospital employee?	3
	Did you talk about the costs of your child's tests and treatments with a hospital employee?	De novo
	Did you know that this hospital has financial counselors?	

©2021 Bassett HK et al. *JAMA Network Open*.

	Did you ask to speak with a financial counselor during your child's hospitalization?	
	Why did you ask to speak with a financial counselor?	
**Perceived cost and quality associations**	A hospital that charges more than another hospital for the same test or treatment is probably providing higher quality care.	4
	A hospital that charges less than another hospital for the same test or treatment is probably providing lower quality care	
	Treatments that work better usually cost more than treatments that don't work as well.	
	Treatments that do not work as well usually cost less than treatments that don't work as well.	
	Tests that are more accurate usually cost more than tests that are less accurate.	
	Tests that are less accurate usually cost less than tests that are more accurate.	
**Financial difficulties**	Do you or your spouse/partner (if applicable) have any current financial burden from medical care? Financial burden could include: difficulty paying or inability to pay medical bills, using personal savings to pay medical bills, difficulty paying for food, gas, or rent because of medical bills, declaring bankruptcy because of medical bills, etc.	5
	Is your current financial burden due to your hospitalized child's medical care?	De novo
	InCharge Financial Distress/Financial Well-being scale (8 questions)	6
**Insurance coverage**	Is your child's health insurance Medicaid?	De novo
	Does your child's insurance plan have a deductible? A deductible is the amount you have to pay for medical services before your child's health insurance starts to pay.	7
	How much is your child's insurance deductible each year?	
	Before to this hospitalization, had your child met their insurance deductible for the year? In other words, had your child's medical bills added up to more than the amount of their deductible?	De novo
	Does your child's insurance plan require a copayment for hospital services? A copayment is a fixed fee you pay when your child receives a certain type of medical service.	7
	Does your child's insurance plan require coinsurance for hospital services? Coinsurance is a percentage of the total hospital charges you are required to pay after you have met your child's insurance deductible.	De novo
**Sociodemographics**	What is your relationship to your child?	De novo
	What is your age?	

©2021 Bassett HK et al. *JAMA Network Open*.

	What is the highest level of education you or your partner/spouse (if applicable) have completed?	
	What is your total yearly household income before taxes?	
	Including yourself, how many people (adults and children) does this household income support?	

©2021 Bassett HK et al. *JAMA Network Open*.

eTable 2. Characteristics of Parent Respondents and Their Hospitalized Children Stratified by Study Site

**Characteristic**	Lucile Packard Children's Hospital Stanford (n=153)	Seattle Children's Hospital (n=74)	Primary Children's Hospital (n=68)	Texas Children's Hospital (n=81)	American Family Children's Hospital (n=75)	Cincinnati Children's Hospital and Medical Center (n=75)
**Financial distress, category**						
High	41 (27)	20 (27)	8 (12)	23 (28)	13 (17)	20 (27)
Average	71 (46)	32 (43)	33 (49)	46 (57)	43 (57)	37 (49)
Low	41 (27)	22 (30)	27 (40)	12 (15)	19 (25)	18 (24)
**Medical financial burden**						
Yes	43 (29)	19 (26)	13 (19)	34 (42)	21 (28)	30 (41)
Related to hospitalized child (% of those with any burden)	27 (63)	4 (21)	9 (70)	23 (68)	10 (48)	13 (43)
No	106 (71)	55 (74)	55 (81)	47 (58)	54 (72)	44 (60)
Missing	4 (3)	0 (0)	0 (0)	0 (0)	0 (0)	1 (1)
**Child's chronic disease level[a]**						
Complex chronic disease	86 (56)	16 (21)	28 (41)	33 (41)	33 (44)	29 (39)
Non-complex chronic disease	24 (16)	58 (78)	10 (15)	18 (23)	23 (31)	10 (13)
No chronic disease	43 (28)	0 (0)	30 (44)	29 (36)	19 (25)	36 (48)
Missing	0 (0)	0 (0)	0 (0)	1 (1)	0 (0)	0 (0)
**Intensive care unit during admission[a]**						
Yes	38 (25)	5 (6)	14 (21)	17 (21)	7 (9)	9 (12)
No	115 (75)	69 (93)	54 (79)	64 (79)	68 (91)	65 (88)
Missing	0 (0)	0 (0)	0 (0)	0 (0)	0 (0)	1 (1)
**Length of stay, days[a]**						
Median (IQR)	4 (2-10)	3.5 (2-7)	4 (2-7)	4 (2-8)	2 (1-5)	2 (1.5-5.5)
Missing	0 (0)	0 (0)	0 (0)	27 (33)	0 (0)	0 (0)
**Child's insurance[a]**						
Public	71 (46)	47 (64)	25 (37)	41 (51)	22 (29)	38 (51)
Private	80 (52)	25 (34)	43 (63)	37 (46)	53 (71)	36 (48)

©2021 Bassett HK et al. *JAMA Network Open*.

High deductible[b] (>$1000/year)	54 (42)	15 (23)	29 (45)	26 (36)	20 (38)	19 (29)
Self-pay	1 (1)	0 (0)	0 (0)	0 (0)	0 (0)	0 (0)
Unknown / Missing	1 (1)	2 (3)	0 (0)	3 (4)	0 (0)	1 (1)
**Ethnicity[a]**						
Not-Hispanic / Latino	96 (63)	59 (80)	56 (82)	50 (62)	67 (89)	72 (96)
Hispanic / Latino	50 (33)	13 (18)	12 (18)	28 (35)	5 (7)	3 (4)
Unknown / Missing	7 (5)	2 (3)	0 (0)	3 (4)	3 (4)	0 (0)
**Race[a]**						
White / Caucasian	68 (44)	51 (69)	63 (93)	61 (75)	62 (83)	57 (76)
Asian	21 (14)	6 (8)	2 (3)	2 (3)	4 (5)	1 (1)
Black / African American	8 (5)	0 (0)	2 (3)	15 (19)	4 (5)	13 (17)
American Indian / Alaska Native	1 (1)	1 (1)	0 (0)	1 (1)	1 (1)	0 (0)
Native Hawaiian / Other Pacific Islander	3 (2)	0 (0)	1 (2)	0 (0)	1 (1)	0 (0)
Other	46 (30)	14 (19)	0 (0)	0 (0)	0 (0)	2 (3)
Unknown / Missing	6 (4)	2 (3)	0 (0)	2 (3)	3 (4)	2 (3)
**% Federal Poverty Level[c]**						
≥400%	55 (41)	22 (33)	20 (31)	22 (31)	21 (29)	17 (25)
200 - 399%	21 (16)	30 (45)	24 (37)	17 (25)	30 (41)	16 (23)
100 - 199%	31 (23)	14 (21)	18 (28)	7 (10)	13 (18)	7 (10)
<100%	26 (20)	1 (2)	3 (5)	24 (34)	9 (12)	29 (42)
Prefer not to answer / Missing	20 (13)	7 (10)	3 (4)	11 (14)	2 (3)	6 (8)
**Parental Educational Level[b] (highest attained by either)**						
Less than high school	7 (5)	2 (3)	2 (3)	7 (9)	1 (1)	4 (5)
High school / GED	42 (29)	21 (28)	20 (29)	27 (34)	17 (23)	29 (40)
Associate’s degree	31 (21)	17 (23)	8 (12)	19 (24)	20 (27)	11 (15)
Bachelor’s degree	29 (20)	22 (30)	28 (41)	19 (24)	24 (32)	11 (15)
Graduate degree	36 (25)	12 (16)	10 (15)	8 (10)	13 (17)	18 (25)
Prefer not to answer / Missing	8 (5)	0 (0)	0 (0)	1 (1)	0 (0)	2 (3)

[a]Abstracted from the electronic health record

©2021 Bassett HK et al. *JAMA Network Open*.

[b]Self reported
[c]Calculated value based on reported annual household income and number of individuals living in the household

eTable 3. Parental Preferences on Knowing, Discussing, and Considering Their Child's Health Care Costs, Stratified by Study Site

Survey measure	Lucile Packard Children's Hospital Stanford (n=153)	Seattle Children's Hospital (n=74)	Primary Children's Hospital (n=68)	Texas Children's Hospital (n=81)	American Family Children's Hospital (n=75)	Cincinnati Children's Hospital and Medical Center (n=75)
**It is important to me to know about the costs of my child's test and treatments.**						
Strongly Agree	80 (52)	24 (32)	34 (50)	51 (63)	29 (39)	40 (53)
Agree	43 (28)	30 (41)	15 (22)	14 (17)	22 (30)	15 (20)
Neither agree nor disagree	14 (9)	6 (8)	8 (12)	9 (11)	13 (17)	9 (12)
Disagree	9 (6)	7 (10)	7 (10)	4 (5)	9 (12)	4 (5)
Strongly disagree	7 (5)	7 (10)	4 (6)	3 (4)	2 (3)	7 (9)
**A hospital employee should talk to me about the costs I will have to pay for my child's tests and treatments.**						
Strongly Agree	79 (52)	31 (42)	33 (49)	43 (53)	26 (35)	33 (44)
Agree	46 (30)	20 (27)	17 (25)	19 (24)	27 (36)	22 (29)
Neither agree nor disagree	11 (7)	9 (12)	8 (12)	10 (12)	15 (20)	7 (9)
Disagree	9 (6)	8 (11)	8 (12)	4 (5)	7 (9)	8 (11)
Strongly disagree	8 (5)	6 (8)	2 (3)	5 (6)	0 (0)	5 (7)
**When choosing a test of treatment for my child, my child's doctor should consider the amount of money it will cost me.**						
Strongly Agree	34 (22)	15 (20)	31 (46)	23 (28)	9 (12)	12 (16)
Agree	36 (24)	26 (35)	17 (25)	21 (26)	22 (29)	12 (16)
Neither agree nor disagree	27 (18)	12 (16)	4 (6)	13 (16)	13 (17)	14 (19)
Disagree	24 (16)	12 (16)	10 (15)	11 (14)	19)25)	14 (19)
Strongly disagree	32 (21)	9 (12)	6 (9)	13 (16)	12 (16)	23 (31)

©2021 Bassett HK et al. *JAMA Network Open*.

**I think about how much it will cost me when I make a decision about my child's tests and treatments.**						
Strongly Agree	34 (22)	12 (16)	19 (28)	23 (28)	11 (15)	8 (11)
Agree	38 (25)	31 (42)	19 (28)	16 (20)	19 (25)	20 (27)
Neither agree nor disagree	22 (14)	12 (16)	13 (19)	13 (16)	13 (17)	11 (15)
Disagree	19 (12)	9 (12)	8 (12)	10 (12)	11 (15)	14 (19)
Strongly disagree	40 (26)	10 (14)	9 (13)	19 (24)	21 (28)	22 (30)

©2021 Bassett HK et al. *JAMA Network Open*.

eReferences.

1. Irwin B, et al. Patient experience and attitudes toward addressing the cost of breast cancer care. *The oncologist*. 2014;19(11):1135-1140.
2. Bullock A, et al. Understanding patient's attitudes toward communication about the cost of cancer care. *J Oncol Pract*. 2012;8(4):e50-58.
3. Alexander GC, Casalino LP, Meltzer DO. Patient-physician communication about out-of-pocket costs. *JAMA*. 2003;290(7):953-958.
4. Phillips KA, Schleifer D, Hagelskamp C. Most Americans do not believe that there is an association between health care prices and quality of care. *Health affairs*. 2016;35(4):647-653.
5. Galbraith AA, et al. Nearly half of families in high-deductible health plans whose members have chronic conditions face substantial financial burden. *Health Affairs*. 2011;30(2):322-331.
6. Prawitz AD, et al. InCharge Financial Distress/Financial Well-being Scale: Development, administration, and score interpretation. *Journal of Financial Planning and Counseling.*2006;17(1):34-50.
7. Schleifer D, Hagelskamp C, Rinehart C. How much will it cost? How Americans use prices in healthcare. *Public Agenda*. 2015. http://www.publicagenda.org/files/HowMuchWillItCost_PublicAgenda_2015.pdf

©2021 Bassett HK et al. *JAMA Network Open*.
